# Next-Generation Whole-Genome Sequencing of Eight Strains of *Bacillus cereus*, Isolated from Food

**DOI:** 10.1128/genomeA.01480-15

**Published:** 2015-12-17

**Authors:** Antonina O. Krawczyk, Anne de Jong, Robyn T. Eijlander, Erwin M. Berendsen, Siger Holsappel, Marjon H. J. Wells-Bennik, Oscar P. Kuipers

**Affiliations:** a Molecular Genetics, University of Groningen, Groningen, the Netherlands; b NIZO food research, Ede, the Netherlands; c Top Institute Food and Nutrition (TIFN), Wageningen, the Netherlands

## Abstract

*Bacillus cereus* can contaminate food and cause emetic and diarrheal foodborne illness. Here, we report whole-genome sequences of eight strains of *B. cereus*, isolated from different food sources.

## GENOME ANNOUNCEMENT

*Bacillus cereus* is a mesophilic or psychrotrophic, spore-forming bacterium commonly present in soil (1). It occurs in the rhizosphere of plants (2, 3) and as a part of animal intestinal microflora (4). It is opportunistically pathogenic and leads to various infections, including: local infections of wounds, or an eye; bacteremia and septicemia; respiratory infections; central nervous system infections; pericarditis; and endocarditis (5). Due to its presence in soil and production of spores, *B. cereus* often contaminates various food products. Consumption of foods with high levels of *B. cereus* may result in two types of foodborne illness: emetic or diarrheal. The emetic type, characterized by vomiting and nausea, is induced by the cereulide toxin produced by cells growing in food (6–8). The diarrheal illness is caused by enterotoxins, including hemolysin BL, cytotoxin K, and nonhemolytic enterotoxin, which are produced by *B. cereus* cells in the small intestine (6, 7). *B. cereus* is closely related to *Bacillus anthracis*, the causative agent of anthrax, and to the insect pathogen, *Bacillus thuringiensis* (9).

Eight strains of *B. cereus*, isolated from different food sources were sequenced by next-generation whole-genome sequencing. The strains were grown at 30°C with shaking at 220 rpm in heart infusion (BHI) broth (Difco). The overnight cultures were diluted in fresh medium to the optical density at 600 nm (OD_600_) and harvested by centrifugation at 5,000 relative centrifugal force (RCF). Subsequently, total DNA was isolated by phenol-chloroform extraction as described previously (10). The isolated DNA was sheared to 500-bp fragments in the Covaris (KBioscience) ultrasone device for preparing the NGS library preps using the paired-end NEB NExtGen library preparation kit. The libraries were 101-base paired-end sequenced on an Illumina HiSeq2000. Subsequently, Velvet (11) was used to perform a *de novo* paired-end assembly on each genome resulting in the draft genome sequences. The RAST server (12) and BAGEL3 (13) were used to annotate the genomes and to identify putative bacteriocin gene clusters, respectively.

### Nucleotide sequence accession numbers.

The genome sequence of the eight *Bacillus cereus* strains have been deposited as whole-genome shotgun projects at DDBJ/EMBL/GenBank under the accession numbers listed in Table 1.

**TABLE 1:** Sequenced strains and their sources*^a^*

*B. cereus strain*	Source	Accession no.
B4077	Chilled dessert	LCYI00000000
B4078	Food, undefined	LCYJ00000000
B4080	Dried onion	LCYK00000000
B4086	Boiled rice	LCYL00000000
B4087	Pea soup	LCYM00000000
B4147	Cereals, pasta and pastries	LCYN00000000
B4153	Dairy products	LCYO00000000
B4158	Vegetables	LCYP00000000

a B-numbers refer to the strain collection at NIZO food research and the University of Groningen (Molecular Genetics).
